# Dicationic ionic liquids (DILs) as rapid esterification catalyst of butyric fatty acid

**DOI:** 10.1038/s41598-023-45851-4

**Published:** 2023-10-30

**Authors:** Raghda. A. El-Nagar

**Affiliations:** Petroleum Testing Lab, Analysis and Evaluation Department, Egyptian Petroleum Research Institute, Nasr City, 11727 Cairo Egypt

**Keywords:** Catalysis, Green chemistry, Chemical synthesis

## Abstract

In the presented work, a series of asymmetric dicationic ionic liquids (ADILs) with different alkyl chain length spacer between the two cation nuclei (imidazolium and pyridinium) with chlorine halide anion was designed, synthesized with excellent yield (89, 90 and 88%) and well characterized via different tools of analysis (FT-IR and ^1^H-NMR spectroscopy and thermal gravimetric analysis; TGA). The synthesized ADILs were examined for potential esterification as recyclable catalysts including the activity of catalytic performance, the reaction conditions justifying. The noted resulted data indicated that the butyric acid was converted perfectly into ester in presence of ADILs with short time of reaction. By completing our studies through the effect of chemical structures, concentrations, time and temperatures, we found that the synthesized Py-6-Imi exhibit the best catalytic performance with 96% as conversion value after 20 min at the ambient temperature (25 °C). The synthesized ADILs also recovered and reused for minimum three rounds without any significant reduction in the catalytic performance. Totally, the usage of ADILs in the esterification process offers lots of benefits such as perfect yield, quick time and environmentally friendly characteristics which make them the optimum sustainable compounds to be achieved in variety of industrial applications.

## Introduction

Recently, catalysts employed in the esterification process have become a popular research area regarding to their great significance for the production efficiency. Strong acids, otherwise inorganic (such as, HCl, H_2_SO_4_ and H_3_PO_4_)^1,2^ or solid acids (such as resins and heteropoly acids), are usually used in catalyses process for the traditional esterification with high catalytic efficiency^3,4^. Unfortunately, the usage of acidic catalysts suffers many disadvantages, including difficult separation from the reaction solution, environmental pollution and equipment corrosion^5^.

Ionic liquids (ILs) are ion salts that mainly consist of positive cations and negative anions and have a melting point below 100 °C^6,7^. ILs were attracted the world attention because of their unique and remarkable chemical and physical properties, such as high stability, low toxicity and volatility^8–10^. The easily separation, recovery and their ability to dissolve different variety of compounds, including many technological and chemical processes make ILs one of the most tremendously selected compounds in esterification processes^11–14^.

ILs with imidazolium cations have been used in successful way as slightly toxic and biodegradable catalysts in esterification processes of acids and alkyl halides under mild conditions^15^. So, ILs with proper cations and anions may be friendly reaction catalysts and media for esters and their derivative synthesis to be further applied in pharmaceutical, medical and industrial applications^16^. Dicationic ionic liquids (DILs), as a new approach of ionic liquids, represent an interesting variation more than the monocationic analogy in terms of properties as well as the tenability of chemical and physical properties^17^.Therefore, they have good potential to be used in many applications^18,19^.

Chinnappan and Kim^20–22^ in their studies used a group of pyridinium dicationic ILs. They synthesized, characterized and evaluated the synthesised DILs as esterification catalysts. Khiratkar et al.^23^ synthesized a sulphonic acid-functionalized benzimidazolium based poly ionic liquid (SAFBPIL) as an esterification catalyst. The resulted data obtained that the carboxylic acids converted successfully into their relative esters with excellent yield and selectivity^24–26^.

In our study, we mainly devoted to synthesize a series of different alkyl chain length spacers between the two cation nuclei (imidazolium and pyridinium) with a chlorine halide anion. These three functionalized asymmetric dicationic ionic liquids (ADILs) Py-2-Imi, Py-6-Imi and Py-10-Imi were characterized and evaluated in Table 1 to convert the carboxylic acids to their relative esters using the typical esterification reaction of butyric acid and benzyl chloride. Their catalytic activity performance was illustrated and the ability of recyclability was explored.

**Table 1:** The chemical structures and physical properties of the synthesized ADILs.

Comp.	Structure	Yield %	Viscosity@ 30 °C (cP)	Density@30 °C (g\cc)	Water content (ppm)
Py-2-Imi		89	2.62	2.07	364
Py-6-Imi		90	2.84	2.12	226
Py-10-Imi		88	3.14	2.23	241

## Experimental

### Materials and methodology

All the used chemicals and reagents were of analytical grade, supplied by Merck, and were used directly without further purifications. 1-Chlorohexane (≥ 98%), 2-methylimidazole (≥ 99%), pyridine (≥ 98%), potassium hydroxide (≥ 97%), acetonitrile (≥ 97%), dichloroethane (≥ 99%), dichlorohexane (≥ 98%), dichlorodecane (≥ 99%), ethyl acetate (≥ 98%), Sodium sulphate (≥ 97%), butyric acid (≥ 99%), benzyl chloride(≥ 99%), triethyl amine (≥ 98%).

#### Synthesis of amphiphilic asymmetric dicationic ionic liquids (ADILs)

**Figure 1:**
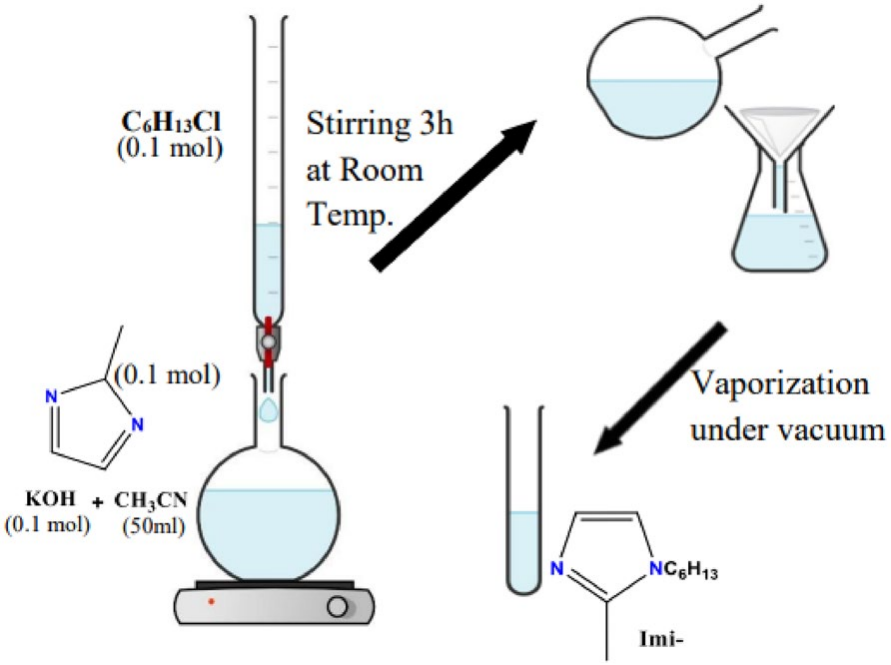
Scale reaction synthesis of 1-hexly-2-methyl imidazole (Imi-).

As shown in Fig. 1, 1-hexly-2-methyl imidazole (Imi-) was prepared by stirring 0.1 mol of 2-methyl imidazole and potassium hydroxide in 50 ml of acetonitrile. 0.1 mol of 1-Chlorohexane was added dropwise to the mixture after complete miscibility. By vagarious stirring for about 3 h, white precipitate of KBr was formed and eliminated by filtration. The filtrate was vaporized and concentrated under vacuum^27^.

**Figure 2:**
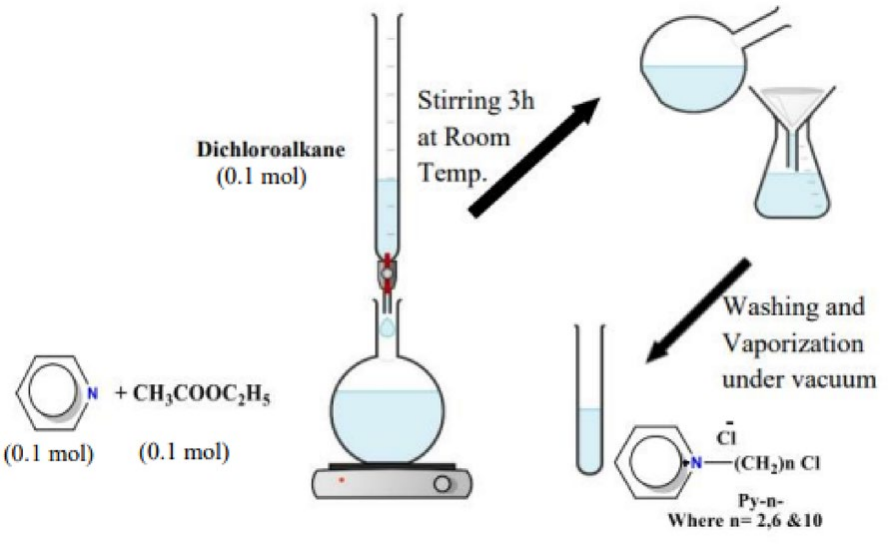
Scale reaction synthesis of 1-(n-Chloroalkyl)-pyridin-1-ium Chloride (Py-n-).

1-(n-Chloroalkyl)-pyridin-1-ium Cloride (Py-n-), Compounds were prepared by stirring 0.1 mol pyridine with 0.1 mol n-di-Chloroalkanes (C_2_, C_6_ and C_10_) at room temperature. Filtrate the white precipitate and wash with ethyl acetate. The filtrate evaporated and was concentrated under vacuum Fig. 2.

**Figure 3:**
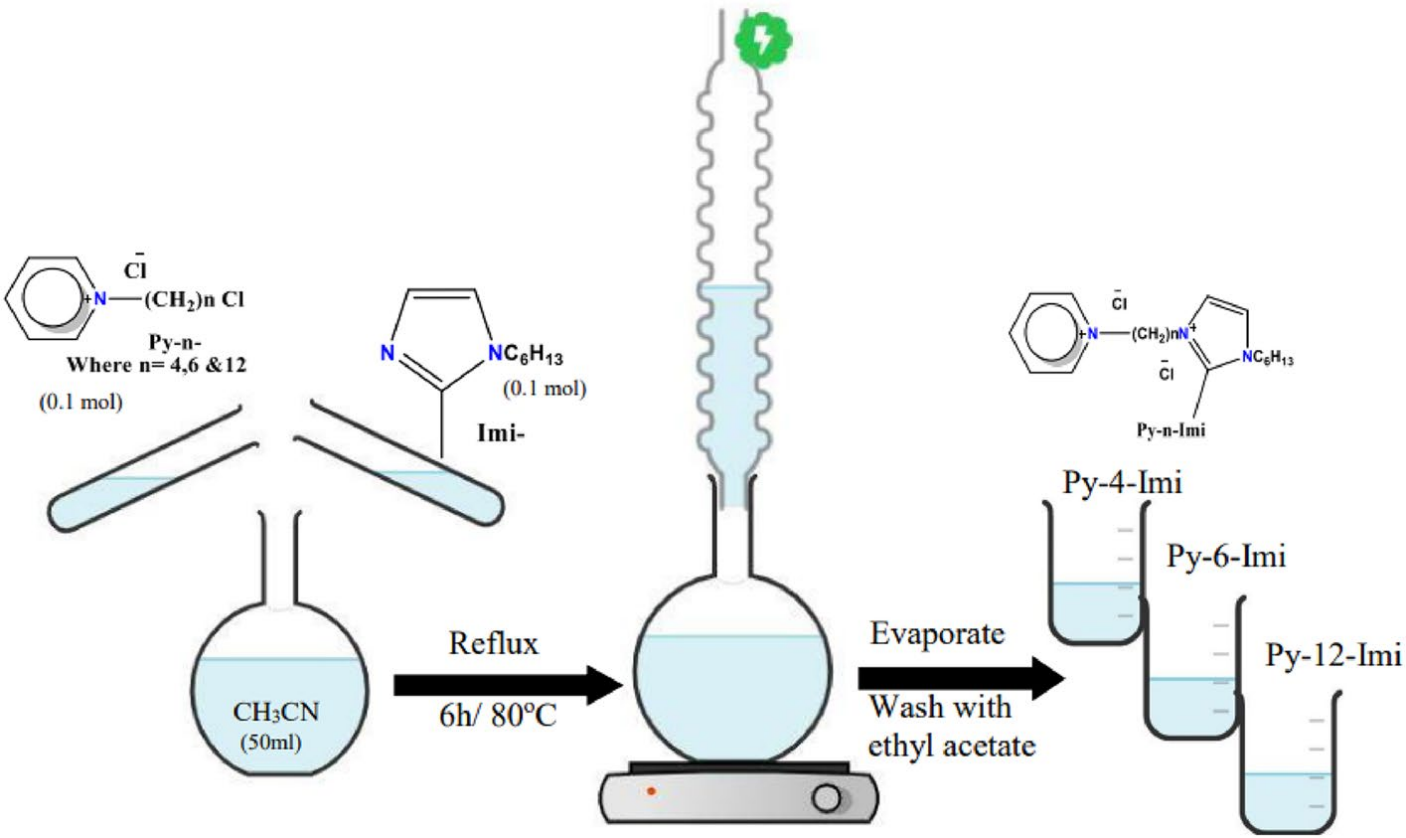
Scale reaction synthesis of 1-(n-(1-hexyl-2-methyl-1H-imidazol-3-ium)-3-hexyl) Pyridinium) Chloride (Py-n-Imi).

1-(n-(1-hexyl-2-methyl-1H-imidazol-3-ium)-3-hexyl) Pyridinium) Chloride (Py-n-Imi) dicationic ionic liquids were synthesized by 3h refluxing of mixtures of compound Py-n- with Imi- in acetonitrile. The products were evaporated and concentrated under vacuum^17^ Fig. 3.

#### Esterification typical procedures

**Figure 4:**
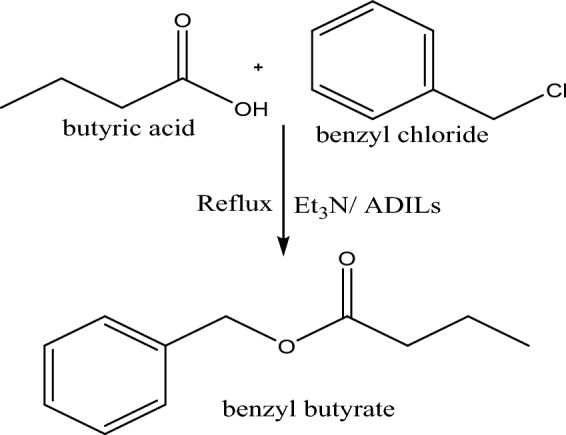
Mechanism of typical esterification.

As a typical esterification process as expressed in Fig. 4, add the prepared ADILs (Py-2-Imi, Py-6-Imi and Py-10-Imi) to a stirring mixture of butyric acid and benzyl chloride (1:1), and then triethyl amine was added at room temperature. The reaction mixture was refluxed for 1h at the ambient temperature (25 °C) in presence of 6 wt. % as ADILs ratio during the main experiment. The formation of a white solid precipitate of Et_3_N.HCl is a perfect indication of the reaction progress, and the reaction completion was detected by TLC^25^. Extract the product using 15 × 3 ml of ethyl acetate and dry it with anhydrous Na_2_SO_4_. The ethyl acetate was evaporated and the product was concentrated under vacuum^26^.

The obtained product concentrations were determined via the liquid chromatographic technique. The samples were collected at different times (2, 5, 10, 15, 20 and 25 min), at different temperatures (25, 35, 45, 55 and 65 °C) and different ADILs dosage to study the influence of time, temperature and dosages on the yield so that, 50 ml of the mixture was sampled and extracted using ethyl acetate. The extracted samples were diluted in acetonitrile and analyzed by the HPLC technique by determining the peak area regarding to the calibration curve method.

#### Ionic liquids recycling process

The importance of ILs recyclability refers to act as sustainable material for other catalysts. After the end of the esterification reaction, the used IL was recovered from the post-reaction filtrate by treating the aqueous layer with ethyl acetate^28^. The solvent was vaporized under vacuum. The structure of the recycled IL was confirmed using FT-IR spectroscopy and reused effectively in new esterification process for 3 times with the same typical esterification process.

#### Characterizations

The **FT-IR** bands of the prepared compounds were examined using a Nicolet Ia-10 at scan resolutions of 4000–400/cm and 4 cm and a scan rate of 32 cm/min, in that order**. **^**1**^**H-NMR** spectra were screened using BURKER ^1^H-NMR spectroscopy in DMSO-d6 solvent. (400.19 MHz and a 5-mm broad-band inverse Z gradient probe). **Thermo-gravimetric analysis (TGA)** was studied using a thermal analyzer at a heating rate of 10 °C/min. Samples are heated from ambient temperature up to 600 °C under nitrogen flow. The thermal degradation discussion was revealed at the point of 95% weight loss from the original weight. **HPLC** (Agilent Technologies 1200 Series HPLC) with a UV detector using a ZORBAX 5 µm, 4.6 × 250 mm NH_2_-column.

## Results and discussion

### Confirmation of the synthesized ADILs

#### FT-IR spectroscopy

**Figure 5:**
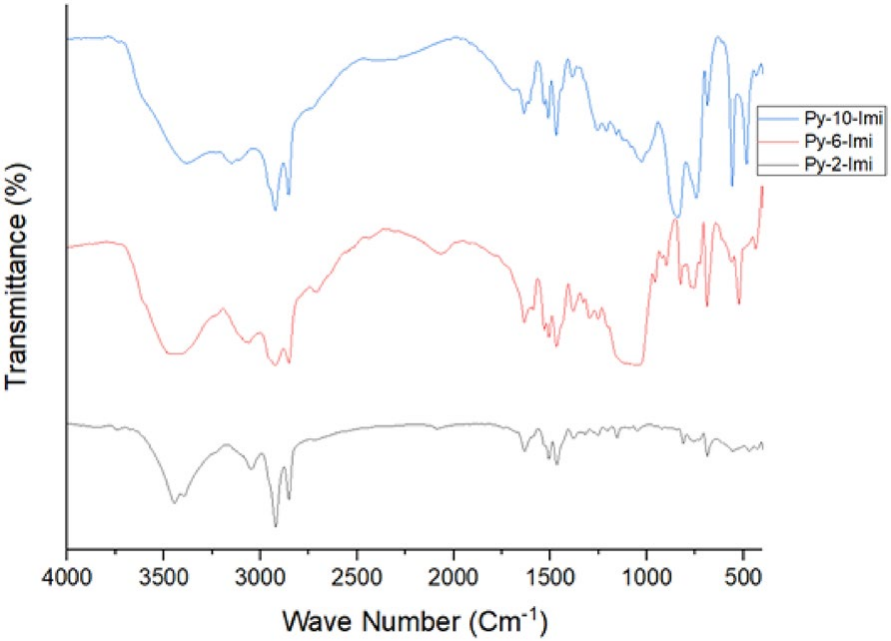
FT-IR spectrum for the synthesized ADILs ^1^H-NMR spectroscopy.

The listed FT-IR spectra in Table 2 and Fig. 5 revealed the featured bands at 3442, 3420 and 3425/cm related for N–H stretching vibrations of Py-2-Imi, Py-6-Imi and Py-10-Imi respectively. Also, this could be attributed to the presence of the carbene proton in the form of NH^+^^29^. The characteristic bands of aromatic C-H stretching bands appeared at 3064, 3054, 3123 and 3011/cm for Py-2-Imi, Py-6-Imi and Py-10-Imi respectively. Aliphatic C–H (stretching) bands appeared at 2848, 2920, 2850, 2923, 2854 and 2920/cm respectively^30^. The featured bands of C–C vibrations in imidazole ring appeared at 1630, 1630 and 1627/cm, while the aromatic C=C stretching bands were noticed at 1523, 1526 and 1473/cm for Py-2-Imi, Py-6-Imi and Py-10-Imi respectively. The spectra of C–N stretching modes of vibrations in imidazole rings were detected at 1398, 1380 and 1323/cm. The vibrational bands of the in plane bending appeared at 1192, 1174 and 1175/cm respectively. Moreover, the values of 775, 770 and 752/cm are attributed to the out of plane bending vibrations for the synthesized compounds respectively. The vibrational values of 670, 671 and 703/cm represented the ring deformation out of plane bending for imidazole rings in the synthesized series, respectively.

**Table 2:** FT-IR spectrum for the synthesized ADILs.

Comp.	ν/cm								
	CH–H_2_O N–H stretching	C-H aromatic stretching	C-H aliphatic stretching	C–C aromatic stretching	C=C Aromatic	C–N stretching	C–H in plane bending	C-H out of plane bending	Ring deformation out of plane
Py-2-Imi	3442	3064	2848 2920	1630	1523	1398	1192	775	670
Py-6-Imi	3420	3054 3123	2850 2923	1630	1526	1380	1174	770	671
Py-10-Imi	3425	3011	2854 2920	1627	1473	1323	1175	752	703

#### ^1^HNMR spectroscopy

**Figure 6:**
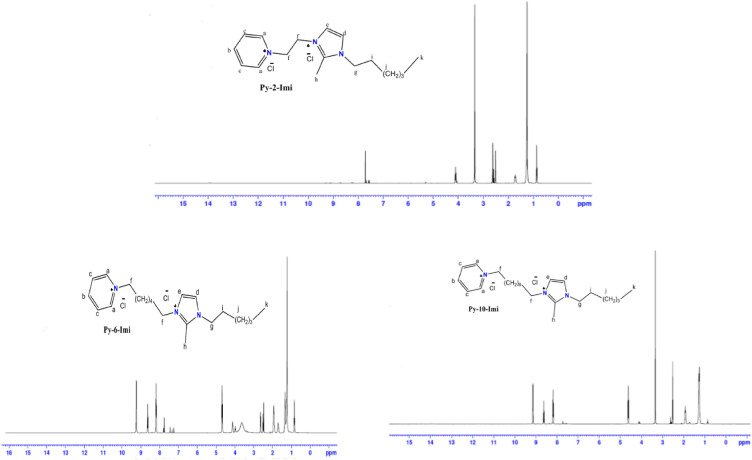
^1^H-NMR Spectroscopy for the synthesized ADILs.

^1^HNMR spectra for the synthesized ADILs and the chemical shifts δ were detected as illustrated in Table 3 and Fig. 6. The highly desheilded protons a, b and c have high δ values because of the drawal action of N. The difference in protons d and e values refers to their direct attachment to N^+^. For Py-2-Imi the four f protons appeared as triplet at δ value 4.18 ppm. For Py-6-Imi and Py-10-Imi protons f appeared different, where f_1_ is highly desheilded more than f_2_ because of the pyridinum and imidazolium ring effects. The triplet proton g is more desheilded than the multiplet proton i due to the effect of aromatic ring. 18 singlet protons j appeared at δ value 1.28, 1.24 and 1.27 ppm respectively, due to their similarity. The aliphatic methyl protons k appeared in the triplet at the lowest δ values.

**Table 3:** ^1^H-NMR Spectra for the synthesized ADILs.

Comp.	a	b	c	d	e	f		g	h	i	j	k
	(d)	(t)	(t)	(d)	(d)	(t)		(t)	(s)	(m)	(s)	(t)
Py-2-Imi	9.13	8.78	8.20	7.63	7.59	4.18		2.67	2.54	1.76	1.28	0.83
Py-6-Imi	9.20	8.69	8.22	7.74	7.27	f_1_	f_2_	3.93	3.66	2.59	1.24	0.82
						4.65	4.22					
Py-10-Imi	9.16	8.59	8.23	7.65	7.58	f_1_	f_2_	2.55	2.58	1.93	1.27	0.85
						4.69	4.19

#### Thermo-gravimetric analysis (TGA)

**Figure 7:**
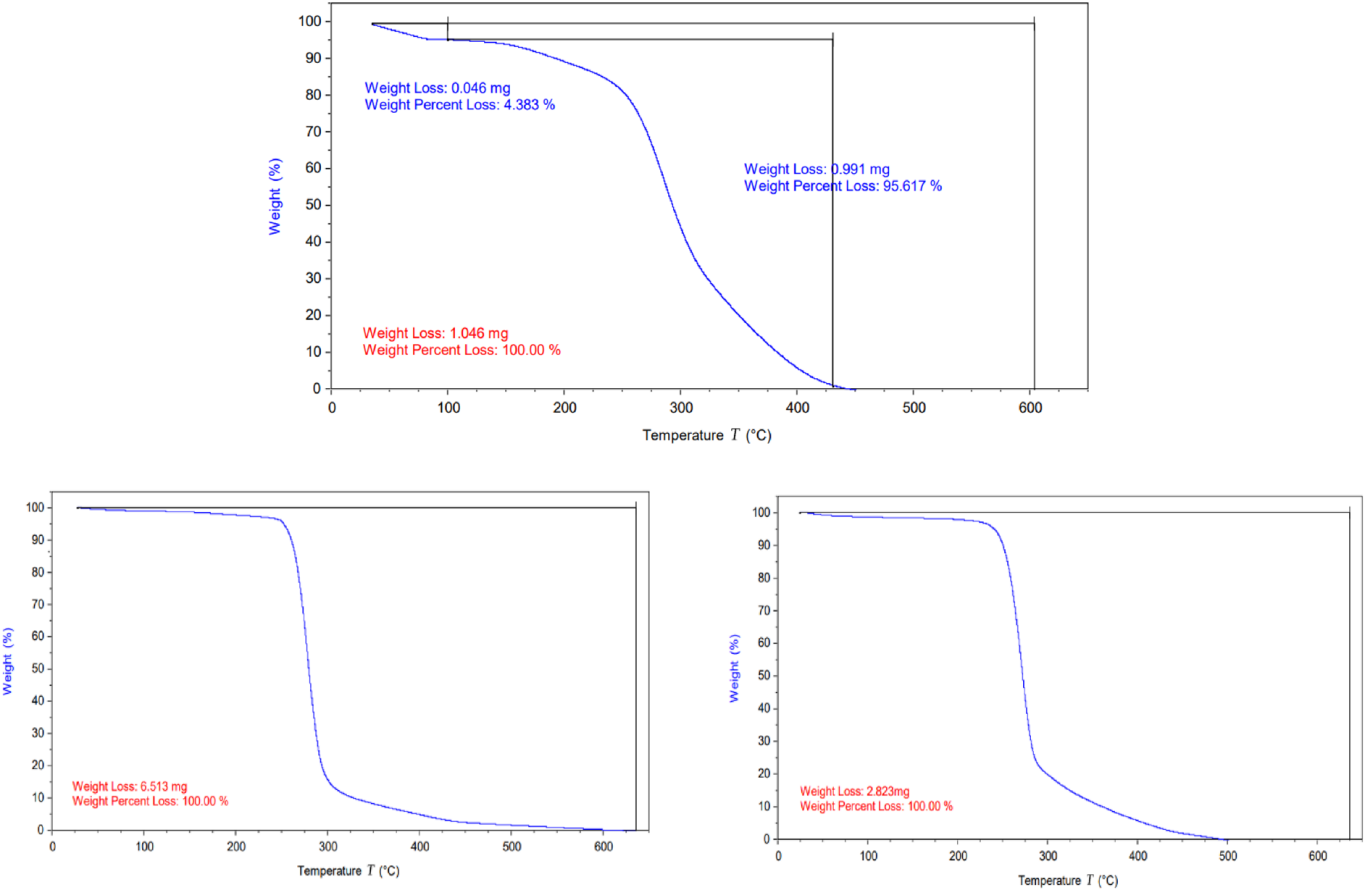
TGA curves for the synthesized ADILs.

TGA curves are experimental studies for the thermal behaviour of the prepared compounds. The synthesized ADILs record high resistance toward the thermal degradation, resulting in high onset temperatures^29,30^. The lower thermal stability was indicated for the longer aliphatic spacer between two cations (imidazolium and pyridinium). Figure 7 confirms that Py-2-Imi, Py-6-Imi and Py-10-Imi are thermally stable and the actually first decomposition steps were noticed at 286, 278 and 270 °C respectively. Higher than these temperatures, TGA indicated an endothermal phenomenon until it reached to maximum mass loss^31,32^.

### Catalytic performance of the synthesized ADILs

**Figure 8:**
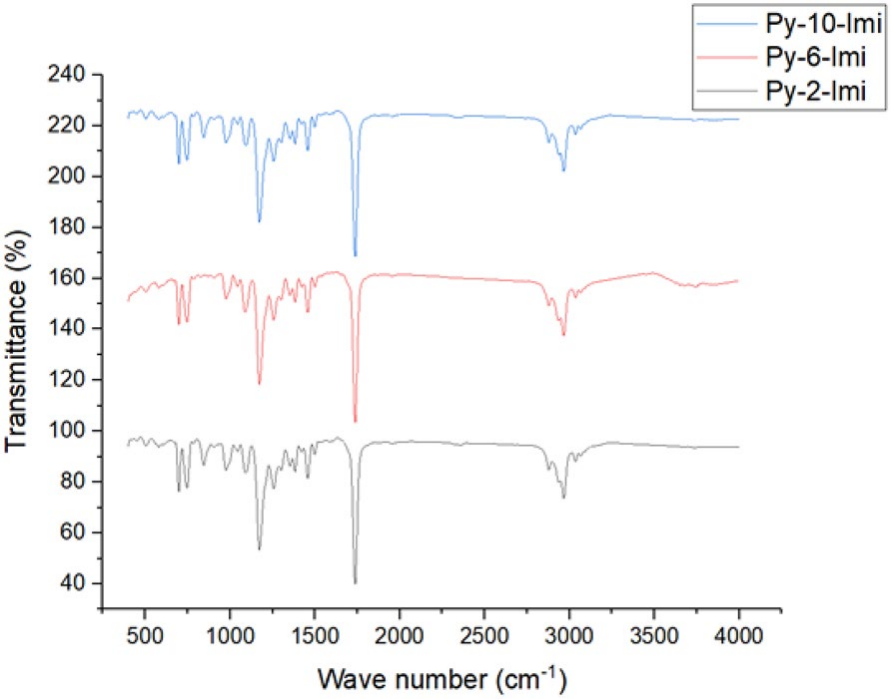
FT-IR of ester using different synthesized ADILs.

The esterification processes of butyric acid and benzyl chloride was carried out using the synthesized ADILs and was studied as catalysts under mild conditions. It was clear that the reaction mixture was successfully converted to an ester (benzyl butyrate) with a good yield. Ester formation was confirmed via FT-IR spectroscopy Fig. 8*.* The carboxylic group characteristic bands were disappeared, and the characteristic carbonyl ester band was observed at 1739/cm.

**Figure 9:**
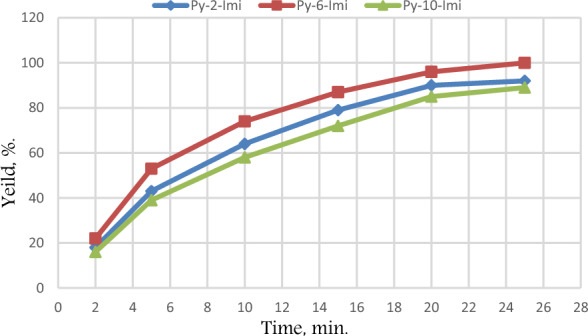
The esterification yield in presence of the synthesized ADILs.

All of the studied ADILs have Cl^-^ as an anion so, the performance activity related directly to the alkyl spacer aliphatic chain length between the two aromatic rings in the cation structure and the effect of moiety. It is well known that aromatic based ILs have high performance activity consequentially, imidazolium cations possessed high activity and acidity^33^ because of the effect of acidic hydrogen connected between the nitrogen atoms in the imidazolium ring. The presence of a pyridinium ring in the cation structure increases the aromaticity. Also, by increasing the length of the alkyl chain between the two aromatic rings, the performance activity, Table 4 increased as shown in Py-6-Imi which indicates higher activity than in Py-2-Imi as the two cation become closer, they act as one cation but by increasing the spacer, the effect of two cations becomes clear. In the case of Py-10-Imi the position of the two cations became far away from each other and that may have happened because of the effect of steric bulk being twisted^34^ and cause decrease in the performance activity Fig. 9.

**Table 4:** The esterification yield in presence of the synthesized ADILs.

Time	Yield (%)		
	Py-2-Imi	Py-6-Imi	Py-10-Imi
2	18	22	16
5	43	53	39
10	64	74	58
15	79	87	72
20	90	96	85
25	92	100	89

#### The influence of ADILs dosage at the esterification processes

**Figure 10:**
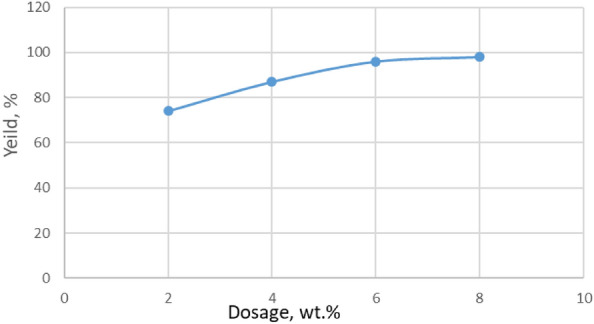
Esterification yields with different dosage of Py-6-Imi.

To complete the evaluation of the synthesized compounds, the effect of temperature was investigated on the esterification reaction yield. Py-6-Imi was selected as the best of the synthesized ADILs for these further studies. The esterification processes were carried out using 2, 4, 6, 8 wt. % of the selected ADIL at the ambient temperature (25 °C). The product was collected after 20 min of the esterification reaction. The obtained data are observed in Fig. 10.

By using 2 wt. %, the ester formation was 74%, whereas with 4 and 6%, the yield increased gradually to reach 87 and 96% respectively. By completing the increase of dose (8 wt. %), the yield started to decrease regarding saturation so, 6 wt. % of Py-6-Imi was considered a relatively efficient dosage for high esterification yields, as shown in Fig. 10.

#### The influence of temperatures at the esterification processes

The effect of temperature was investigated on the esterification reaction yield through carrying out the experiment between 25 and 65 °C temperature ranges by increasing 10 °C in each step. The resulting data are presented in Table 5.

**Table 5:** The esterification yield in presence of Py-6-Imi at different temperatures.

Time	Yield (%)				
	25 °C	35 °C	45 °C	55 °C	65 °C
2	22	29	39	44	64
5	53	65	58	62	100
10	74	84	88	91	
15	87	92	100	100	
20	96	100			
25	100				

**Figure 11:**
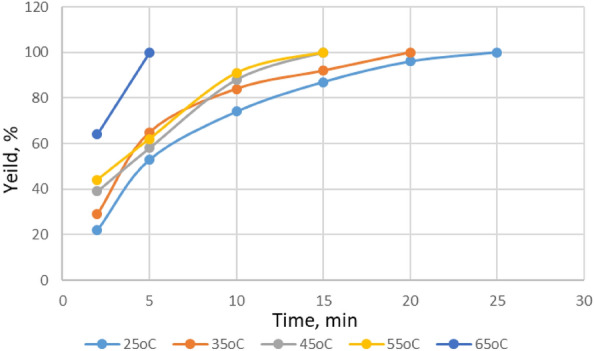
The esterification yield in presence of Py-6-Imi at different temperatures.

The temperature`s considerable impact on the esterification yield in the presence of Py-6-Imi was studied and observed, as in Fig. 11. At the ambient temperature (25 °C), it was noticed that the total conversion into the ester was recorded after 25 min. By increasing the temperature, the time of esterification decreased^35^ to reach the maximum conversion, which reached 5 min at 65 °C. The revealed data illustrate that the use of ADILs in esterification process at mild conditions (temperature range 25–45 °C) enables for significant ester yield to be collected at the same reaction conditions. From the point of using energy to supply heat or remove it, the usage of ADILs as sustainable materials is completely in line with green chemistry approach.

### Recycling

**Figure 12:**
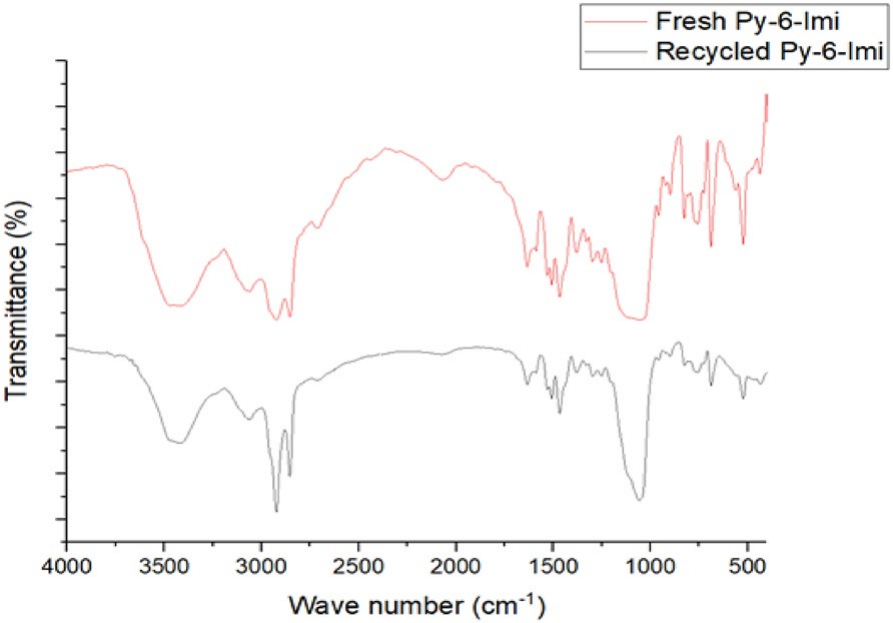
FT-IR for the new and recycled Py-6-Imi.

Py-6-Imi was selected to evaluate the recyclability of ADILs.The experiments on recycling ADILs were carried out at 25 °C, 6wt. % as the dosage concentration and the ester was collected after 20 min after the esterification reaction. When the reaction was completed, the aqueous layer was treated with ethyl acetate to extract the used Py-6-Imi. The solvent was evaporated and dried under a vacuum. The chemical structure of the recyclable Py-6-Imi was confirmed by using FT-IR and there was no noticeable change in its chemical composition^36^ followed by the 1st, 2nd and 3rd recycles as shown in Fig. 12.

**Figure 13:**
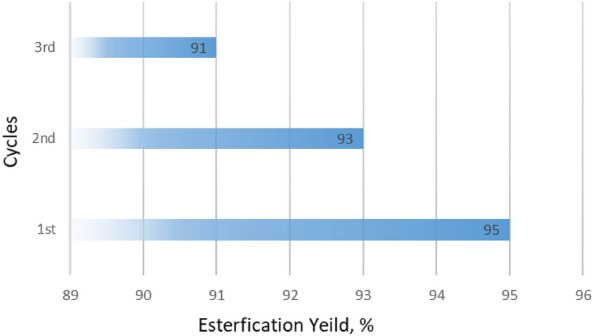
Esterification yield obtained; % with recycled Py-6-Imi.

The recycled Py-6-Imi was involved in the esterification process and the recycling was repeated for three cycles at the optimum mild conditions^37^. The product was collected after 20 min of the esterification process as illustrated in Fig. 13. It was clear that the recycled Py-6-Imi has almost nearly catalytic performance (91, 93 and 95% as esterification yield) without significant change in its chemical composition. From the resulted data, the product yield slightly decreases (maximum decrease 5%) which is still more than 90%, so we were confirmed that the catalyst is completely active.

## Conclusions

In conclusion, this study affords DILs as a new approach for a better conversion in the esterification process. The newly synthesized series were suggested to be a suitable replacement for the conventional catalysts. According to the resulted data, the optimum conditions were found to be 6 wt. % concentration, after 20 min of reaction and at the ambient temperature (25 °C) and the high conversion value reported was 96% for Py-6-Imi compound. The recyclability of the used IL to be recovered and re-entered in a new reaction at least for three times without any significant reduction in the catalytic performance enhances their potential for eco-friendly and cost-saving issues.

## Data Availability

The data that support the findings in the present study are available from the corresponding author upon request.
